# Supplementary material for: A Novel Bio-Inspired Ag/3D-TiO2/Si SERS Substrate with Ordered Moth-like Structure
Source: Nanomaterials (Basel). 2022 Sep 9;12(18):3127. doi: 10.3390/nano12183127 (PMC9501013; doi:10.3390/nano12183127)
Supplement: Supplementary file 1 [file nanomaterials-12-03127-s001.zip › nanomaterials-1872012-supplementary.pdf]

Supplementary Materials

A Novel Bio-inspired Ag/3D-TiO_2/Si SERS Substrate with Ordered Moth-like Structure

Jingguo Yang [1,†], Florian Ion Tiberiu Petrescu [2,†], Ying Li [1], Dandan Song [1] and Gang Shi [1,*]

[1] The Key Laboratory of Food Colloids and Biotechnology, Ministry of Education, School of Chemical and Material Engineering, Jiangnan University, Wuxi 214122, China
[2] Department of Mechanisms and Robots Theory, Bucharest Polytechnic University, Bucharest, Romania
* Correspondence: gangshi@jiangnan.edu.cn; shigang0720@126.com
† These authors contributed equally to this work

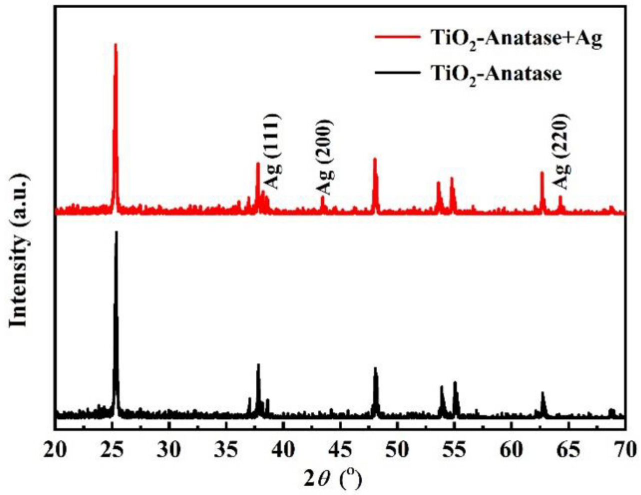

Figure S1. XRD of TiO_2 and TiO_2 deposited with Ag NPs.

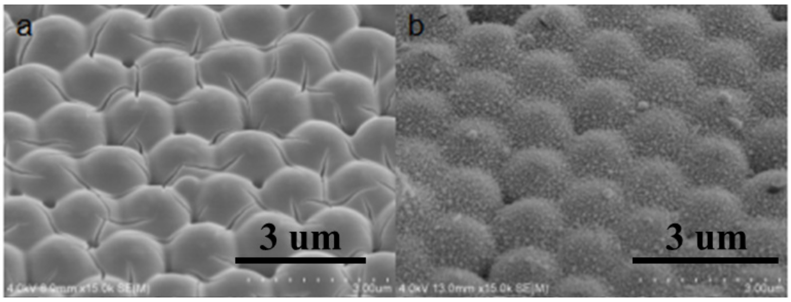

Figure S2. SEM images of (a) 2D-TiO_2/Si and (b) Ag/2D-TiO_2/Si.
